# Human 3*β*‐hydroxysteroid dehydrogenase type 1 in human breast cancer: clinical significance and prognostic associations

**DOI:** 10.1002/cam4.708

**Published:** 2016-05-03

**Authors:** Toru Hanamura, Tokiko Ito, Toshiharu Kanai, Kazuma Maeno, Yasuyo Shimojo, Takeshi Uehara, Takashi Suzuki, Shin‐ichi Hayashi, Ken‐ichi Ito

**Affiliations:** ^1^ Division of Breast and Endocrine Surgery, Department of SurgeryShinshu University School of Medicine3‐1‐1 AsahiMatsumotoNaganoJapan; ^2^ Department of Laboratory MedicineShinshu University HospitalMatsumotoNaganoJapan; ^3^ Department of Pathology and HistotechnologyTohoku University Graduate School of MedicineSendaiMiyagiJapan; ^4^ Center for Regulatory Epigenome and Diseases, Department of Molecular and Functional DynamicsTohoku University Graduate School of MedicineSendaiMiyagiJapan

**Keywords:** 3*β*‐hydroxysteroid dehydrogenase type 1, breast cancer, clinical significance, prognosis, steroid metabolism

## Abstract

Active sex steroids including estrogens and androgens are locally produced from circulating inactive steroids by various steroid‐metabolizing enzymes, and play pivotal roles in the progression of hormone‐dependent breast cancers. Human 3*β*‐hydroxysteroid dehydrogenase type 1 (3*β*‐HSD type 1) is a critical enzyme in the formation of all classes of active steroid hormones, and is also involved in the inactivation of potent androgen dihydrotestosterone (DHT). Therefore, this enzyme is suggested to modulate active sex steroid production or inactivation, with a role in hormone‐dependent breast cancer. The purpose of this study was to investigate the clinical significance of 3*β*‐HSD type 1 in human breast cancer. Using immunohistochemistry (IHC), we evaluated 3*β*‐HSD type 1 expression in 161 human breast cancers and analyzed correlations of 3*β*‐HSD type 1 expression with various clinicopathological factors. Of 161 breast cancer cases, 3*β*‐HSD type 1 expression in cancer cells was detected in 119 cases (73.9%), and was positively correlated with estrogen receptor (ER)‐positivity but not HER‐2 status. In ER‐positive cases (*n* = 130), 3*β*‐HSD type 1 expression was inversely correlated with invasive tumor size (*P *=* *0.0009), presence of invasive region (*P *=* *0.0107), and lymphatic involvement (*P *=* *0.0004). 3*β*‐HSD type 1 expression was significantly associated with decreased risk of recurrence or improved prognosis by both univariate (*P *=* *0.0003 and *P *=* *0.009, respectively) and multivariate (*P *=* *0.027 and *P *=* *0.023, respectively) analyses. Our findings indicate that this enzyme is a prognostic factor in hormone‐dependent breast cancer.

## Introduction

Breast carcinoma is one of the most common malignancies in women worldwide, and the majority of cases are hormone‐dependent 1, 2. In hormone‐dependent breast cancers, estrogens play crucial roles in cancer progression through the nuclear estrogen receptor (ER) 1, 3. Other bioactive sex steroids androgens, such as dihydrotestosterone (DHT) and its precursor testosterone (TS), have been reported to have inhibitory effects in hormone‐dependent breast cancer cells 4, 5, 6. Consequently, it is postulated that the progression of hormone‐dependent breast cancers is regulated by the overall effect of bioactive sex steroids. These active sex steroids are produced in endocrine organs including the ovary in females and the testis in males, and are secreted into the circulation. However, the serum concentration of hormones does not necessarily reflect the local hormonal activities in the target tissues. Numerous recent studies reported that active sex steroids are also locally produced from circulating inactive steroids; this system of hormone action is known as the intracrine system 5, 7, 8, 9, 10. It is also reported that a substantial proportion of estrogens in women (approximately 75% before menopause, and almost 100% after menopause) are synthesized in peripheral hormone‐target tissues from abundant circulating precursor steroids of adrenal origin 5, 10. It is thus important to evaluate the physiological and/or pathological significance of this intracrine activity 7.

As mentioned above, bioactive sex steroids are locally produced from circulating inactive steroids including dehydroepiandrosterone sulfate (DHEAS), dehydroepiandrosterone sulfate (DHEA), and androstenedione (A‐dione) by the comprehensive action of the various steroid‐metabolizing enzymes 5, 10. In estrogen metabolism, aromatase catalyzes the final and rate‐limiting step in the biosynthesis of estrogen from adrenal androgens (TS and A‐dione)^8^. Steroid sulfatase (STS) metabolizes estrone sulfate (E1S) and DHEAS to E1 and DHEA, respectively, which are further metabolized to E2 or androst‐5‐ene‐3*β*,17*β*‐diol (A‐diol), respectively, by 17*β*‐hydroxysteroid dehydrogenase type 1 (17*β*‐HSD type 1)^8^, ^11^, ^12^. In androgen metabolism, DHT is synthesized from TS in an irreversible reaction catalyzed by the 5*α*‐reductase family, and is a highly potent androgen with inhibitory effects in hormone‐responsive breast cancer cells 4, 5, 13.

Human 3*β*‐HSD type 1 is a critical enzyme in the conversion of DHEA and A‐diol to estrogen precursors A‐dione and TS. This enzyme is also involved in conversion of the potent androgen DHT to its inactive form 5*α*‐androstane‐3*β*,17*β*‐diol (3*β*‐diol), which has substantial estrogenic activity 14, 15, 16, 17, 18, 19, 20. Therefore, this enzyme is suggested to be a modulator of active sex steroid production or inactivation, and plays a role in hormone‐dependent breast cancer. However, the expression of 3*β*‐HSD type 1 has not been sufficiently examined in human breast cancer tissue, and the biological significance of 3*β*‐HSD type 1 remains unknown.

The purpose of this study was to examine the expression of 3*β*‐HSD type 1 and its biological and prognostic significance in human breast cancer. To accomplish this, we evaluated the expression of 3*β*‐HSD type 1 in 161 human breast cancer specimens by immunohistochemistry (IHC) and analyzed for correlations with various clinicopathological factors. Our results indicate for the first time that this enzyme is a potent prognostic factor in hormone‐dependent breast cancer.

## Materials and Methods

### Tumor samples

All human breast cancer tissues were obtained during surgery at the Shinshu University Hospital (Nagano, Japan) after patient consent and with approval from the Shinshu University Hospital Ethics Committee. Tumor samples were obtained from all patients with untreated breast cancer who had undergone surgery during January 2004 and December 2005 (*n* = 161). Clinicopathological data including age, menopausal state, invasive tumor size, histological type, lymphatic involvement, lymph node status, histological grade, ER status, progesterone receptor (PgR) status, human epidermal growth factor receptor‐2 (HER‐2) status, and prognosis were collected by reviewing patient case records. At the time of surgery, patients who had not menstruated for more than 1 year were defined as postmenopausal; other patients were defined as premenopausal. The histological grade of each specimen was evaluated by one pathologist (T. U.) based on the method of Robbins et al. 21. ER, PgR, and HER‐2 statuses were evaluated by IHC staining. The cut‐off value for ER and PgR positivity was set at ≥10% 22. Tumors were considered to overexpress HER‐2 if they were given a score of 3 following IHC staining, or if they showed >2.2‐fold amplification of the *HER‐2* gene, as assessed by fluorescence in situ hybridization. FISH testing was only performed for tumors that scored 2 during IHC staining 23. Because adjuvant therapies were administered based on criteria in 2004, it should be noted that the criteria for ER‐, PgR‐, and HER‐2 positivity are not new. None of the patients examined in our study received irradiation, chemotherapy, or endocrine therapy prior to surgery. Sixty‐four patients received adjuvant chemotherapy, while 128 patients received adjuvant endocrine therapy after the surgery. The mean follow‐up time was 104 months (range 4–132 months). Disease‐free and disease‐specific survival data were available for all patients. All specimens were fixed with 10% formalin and embedded in paraffin wax. Snap‐frozen tissues were not available for examination in this study. Patient characteristics are listed in Table 1.

**Table 1:** Clinical and histopathological characteristics of 161 breast cancers

	No. of patients (%)
Age; median (range)	54 (26–82)
Menopausal status (%)	
Premenopausal	67 (41.6)
Postmenopausal	93 (57.8)
Male	1 (0.6)
‘Invasive tumor size (mm; median (range))	15 (1–95)
Histological type (%)	
IDC^a^	116 (72.0)
DCIS^b^	28 (17.4)
ILC^c^	6 (3.7)
LCIS^d^	1 (0.6)
Special type	10 (6.2)
Lymphatic involvement (%)	
Positive	88 (54.7)
Negative	73 (45.3)
Lymph node metastasis (%)	
Positive	61 (37.9)
Negative	99 (61.5)
Unknown	1 (0.6)
Histological grade (%)	
1	39 (24.2)
2	74 (46.0)
3	16 (9.9)
Unknown	32 (19.9)
ER status (%)	
Positive	130 (80.7)
Negative	31 (19.3)
PgR status (%)	
Positive	109 (67.7)
Negative	52 (32.3)
HER‐2 overexpression (%)	
Positive	46 (28.6)
Negative	86 (53.4)
Unknown	29 (18.0)

a Invasive ductal carcinoma.

b Ductal carcinoma in situ2.

c Invasive lobular carcinoma3.

d Lobular carcinoma in situ4.

### Antibodies

Mouse monoclonal antibody for 3*β*‐HSD type 1 (Ab55268) was purchased from Abcam (Cambridge, UK). This antibody was raised against the recombinant full‐length protein, corresponding to amino acids 1–374 of human HSD3B1. Mouse IgG1‐kappa monoclonal antibody (ab18447) for isotype control was purchased from Abcam. Monoclonal antibodies for ER (SP1), PgR (1E2) and HER‐2 (4B5) were purchased from Ventana (Tucson, AZ, USA).

### Immunohistochemistry

A Vectastain ABC kit (VectorLaboratories, Burlingame, CA, USA), which uses the avidin‐biotin complex (ABC) method, was used for the 3*β*‐HSD type 1 immunoreactive staining. After deparaffinization, antigen retrieval was performed by heating the slides in a microwave oven twice for 6 min in a citric acid buffer. The dilution of 3*β*‐HSD type 1 primary antibody was 1:200. After overnight incubation at 4°C with the primary antibody, incubation with the biotinylated anti‐mouse IgG (Vectastain ABC kit) was performed for 1 h at RT. Then, sections were incubated with avidin‐biotin‐peroxidase complex (Vectastain ABC Kit) at RT for 1 h. Finally, the antigen‐antibody complex was visualized after a 6‐min incubation with diaminobenzidine. Counterstaining was performed using hematoxylin. Tissue sections of human placenta 15, human adrenal gland (zona glomerulosa)^24^, and a cell block of HSD3B1‐overexpressing breast cancer cell line (E10‐HSD3B1) and its control cell line (E10‐control), which were established from the MCF‐7 human breast cancer cell line 25, were used as positive controls for 3*β*‐HSD type 1. As a negative control, mouse IgG1‐kappa monoclonal antibody (ab18447) was used instead of the primary antibodies for isotype control. Immunoreactive staining of ER, PgR, and HER‐2 were performed as previously described 26.

### Scoring of immunoreactivity

For statistical analyses of 3*β*‐HSD type 1, the cancer samples were independently and blindly classified into two groups (positive and negative cancer cells) by three of the authors (T. H., T. S., and T. U.). Because there are no reports describing the evaluation criteria of 3*β*‐HSD type 1 in breast cancer in the past, we used the evaluation criteria by Suzuki et al., which reported other steroid‐metabolizing enzymes, with some modifications 27, 28.The cases were defined as positive if more than 50% cancer cells showed cytosolic staining regardless of its intensity; others were defined as negative. Interobserver differences were 11.8% (19 cases). Cases with discordant results among the observers were simultaneously reevaluated using a multiheaded microscope.

### Statistical analysis

Statistical analyses were performed using the StatFlex 6.0 software program (Artech Co., Ltd., Osaka, Japan). Values for patient age and invasive tumor size were summarized as the median (range). Other patient characteristics were expressed in absolute numbers (%). Quantitative data and categorical data were compared using unpaired t‐tests and chi‐square tests, respectively. Disease‐free and disease‐specific survival curves were generated according to the Kaplan–Meier method. The statistical significance of differences in the survival analyses were calculated using the log‐rank test. In addition, univariate and multivariate analyses for prognoses were evaluated by a proportional hazard model (Cox). Significant variables evaluated by univariate analyses were only examined in the multivariate analyses. *P*‐values of <0.05 were considered significant.

## Results

### Immunohistochemistry

**Figure 1:**
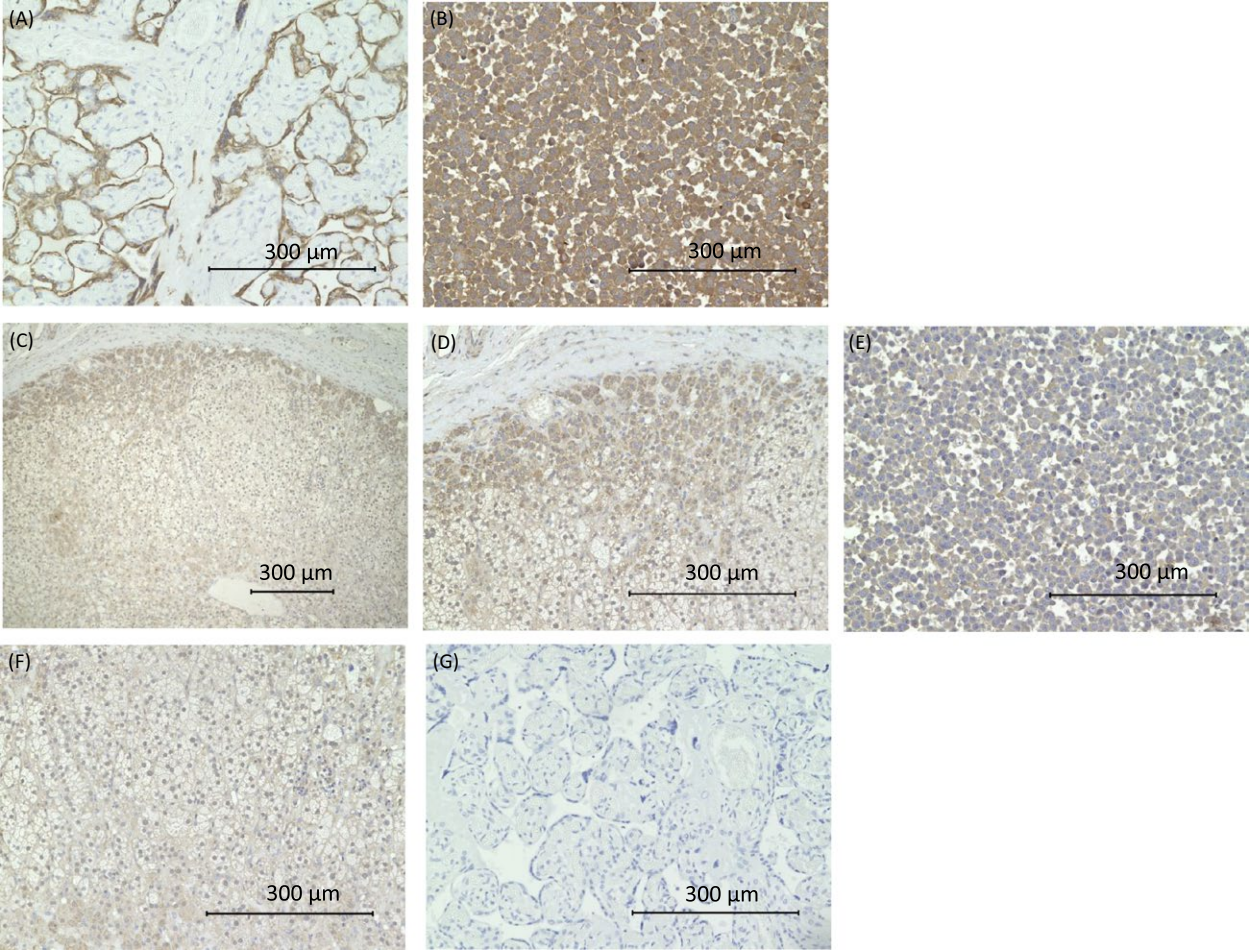
Immunoreactivity of 3*β*‐HSD type 1 in control specimens**.** Strong immunoreactivity of 3*β*‐HSD type 1 was detected in the syncytiotrophoblast of human placenta (A) and the cytoplasm of E10‐HSD3B1 cells (B). Moderate immunoreactivity was detected in the zona glomerulosa of human adrenal glands (C, D) and the cytoplasm of E10‐control cells (E). Immunoreactivity for 3*β*‐HSD type 1 was absent in the zona fasciculata of human adrenal gland (C, F). No immunoreactivity was identified in specimens by isotype control antibodies using standard immunohistochemistry (G). Original magnifications were ×200 (A, B, D, E, F, G) and ×100 (C). Bars indicate 300 *μ*m.

**Figure 2:**
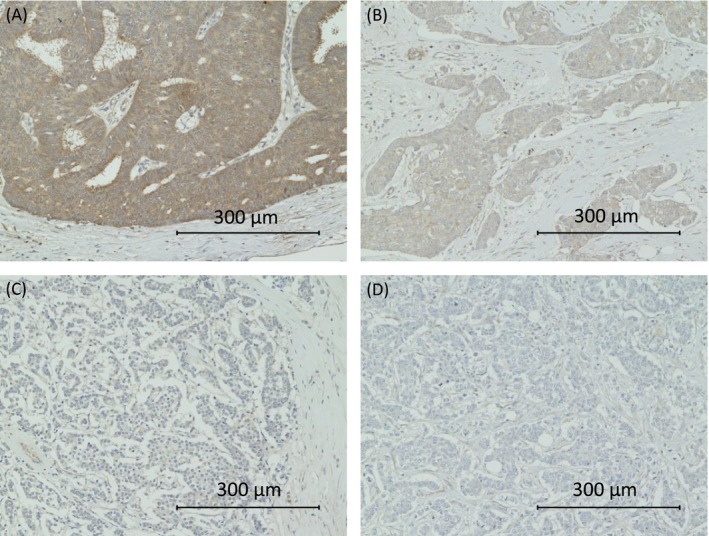
Immunoreactivity of 3*β*‐HSD type 1 in breast cancer specimens**.** 3*β*‐HSD type 1 immunoreactivity was detected in the cytoplasm of breast cancer cells. Typical cases evaluated as positive (A, B) or negative (C, D) are shown.

Strong immunoreactivity of 3*β*‐HSD type 1 was detected in the syncytiotrophoblast of human placenta and the cytoplasm of E10‐HSD3B1 cells (Fig. 1A, B), whereas moderate immunoreactivity was detected in the zona glomerulosa of human adrenal glands and the cytoplasm of E10‐control cells (Fig. 1C, D, E). Immunoreactivity for 3*β*‐HSD type 1 was absent in the zona fasciculata of human adrenal gland (Fig. 1C, F). No immunoreactivity was identified in specimens using isotype control antibodies by standard immunohistochemistry (Fig. 1G). 3*β*‐HSD type 1 immunoreactivity was detected in the cytoplasm of breast cancer cells. The number of cases evaluated as 3*β*‐HSD type 1‐positive and ‐negative were 119 (73.9%) and 42 (26.1%), respectively. Figure 2 shows the typical cases evaluated as positive (Fig. 2A, B) or negative (Fig. 2C, D). Of 118 available cases that had morphologically normal glands adjacent to the cancer tissue, 112 cases (94.9%) had moderate (56 cases; 47.5%) or strong (56 cases; 47.5%) immunoreactivity for 3*β*‐HSD type 1 in morphological normal epithelial cells (data not shown). Moderate (43 cases; 26.7%) or strong (16 cases; 9.9%) immunoreactivity of 3*β*‐HSD type 1 was also detected in stromal cells adjacent to the cancer tissue (data not shown). As mentioned above, cytosolic staining of cancer cells was analyzed in detail in this study.

### **Correlation between 3** *β* **‐HSD type 1 expression and breast cancer subtype**

**Figure 3:**
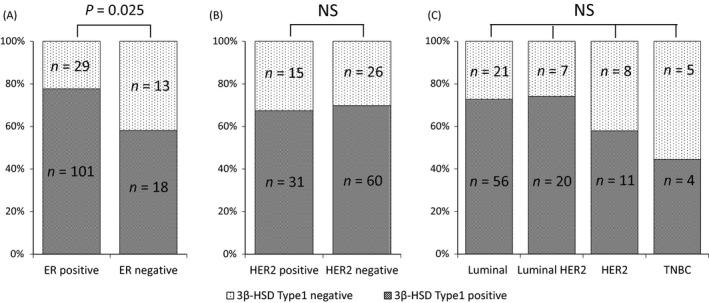
Correlation between 3*β*‐HSD type 1 expression and breast cancer subtype**.** Proportion of 3*β*‐HSD type 1‐positive or ‐negative cases are shown according to ER status (A), HER‐2 status (B), and breast cancer subtype (C), which was approximated as follows: luminal (ER‐positive and HER‐2 negative), luminal HER‐2 (ER‐positive and HER‐2‐positive), HER‐2 (ER‐negative and HER‐2‐positive), and TNBC (ER‐negative and HER‐2‐negative). Absolute numbers of cases in each category are shown inside each graph. Data were compared using chi‐square test. Significance was set as *P *<* *0.05.

First, we analyzed 3*β*‐HSD type 1 expression according to ER or HER‐2 status. The number of cases expressing immunoreactive 3*β*‐HSD type 1 according to ER and HER‐2 status is summarized in Figure 3. Expression of 3*β*‐HSD type 1 was positively correlated with ER‐positivity (Fig. 3A) but not with HER‐2 status (Fig. 3B). For further analysis, using the ER and HER‐2 status of the tumor, breast cancer subtypes were approximated as follows: “luminal” (ER‐positive and HER‐2‐negative), “luminal HER‐2” (ER‐positive and HER‐2‐positive), “HER‐2” (ER‐negative and HER‐2‐positive), and “TNBC” (ER‐negative and HER‐2‐negative). Cases evaluated as 3*β*‐HSD type 1‐negative were most common in TNBC samples without statistical significance (Fig. 3C). Because of these results, we hypothesized that 3*β*‐HSD type 1 has some significant role especially in hormone‐dependent breast cancers.

### Correlation between 3*β*‐HSD type 1 expression and clinicopathological parameters in 130 ER‐positive breast cancers

Table 2 summarizes the identified associations between 3*β*‐HSD type 1 expression and clinicopathological parameters in 130 ER‐positive breast cancers. Of 130 ER‐positive cases, the number of cases evaluated as 3*β*‐HSD type 1‐positive and ‐negative were 101 (77.7%) and 29 (22.3%), respectively. 3*β*‐HSD type 1 expression was inversely correlated with invasive tumor size (*P *=* *0.029), presence of invasive region (*P *=* *0.0107),w and lymphatic involvement (*P *=* *0.0004). There was, however, no significant relationship between 3*β*‐HSD type 1 expression and patient age, menopausal status, lymph node metastasis histological grade, ER status, or HER‐2 status.

**Table 2:** Clinical and histopathological characteristics according to 3*β*‐HSD type 1 status in 130 ER‐positive breast cancers

		3β‐HSD type 1 expression		
	Total	Negative	Positive	
	*n* = 130	*n* = 29	*n* = 101	*P*‐value
Age; median (range)	53.5 (26–82)	56 (31–81)	52 (26–82)	NS
Menopausal status (%)				
Premenopausal	58 (44.6)	9 (31.0)	49 (48.5)	NS
Postmenopausal	71 (54.6)	20 (69.0)	51 (50.5)	
Male	1 (0.8)	0 (0)	1 (1.0)	
Invasive tumor size^a^(mm; median (range))	15.0 (1–95)	18.5 (1–95)	15 (1–56)	*P *=* *0.0292
Histological type (%)				
IDC^b^	92 (70.8)	26 (89.7)	66 (65.3)	*P *=* *0.0107
DCIS^c^	25 (19.2)	1 (3.4)	24 (23.8)	
ILC^d^	5 (3.8)	2 (6.9)	3 (3.0)	
LCIS^e^	1 (0.6)	0 (0)	1 (1.0)	
Special type	10 (6.2)	0 (0)	7 (6.9)	
Lymphatic involvement (%)^a^				
Positive	70 (67.3)	24 (85.7)	46 (60.5)	*P *=* *0.0151
Negative	34 (32.7)	4 (14.3)	30 (39.5)	
Lymph node metastasis (%)^a^				
Positive	48 (46.2)	15 (53.6)	33 (43.3)	NS
Negative	55 (52.9)	12 (42.9)	43 (56.6)	
Unknown	1 (1.0)	1 (3.6)	0 (0)	
Histological grade (%)^a^				
1	36 (27.7)	11 (37.9)	25 (24.8)	NS
2	58 (44.6)	14 (48.3)	44 (43.6)	
3	9 (6.9)	3 (10.3)	6 (5.9)	
Unknown	27 (20.8)	1 (3.4)	26 (25.7)	
PgR status (%)				
Positive	109 (83.8)	25 (86.2)	84 (83.2)	NS
Negative	21 (16.2)	4 (13.8)	17 (16.8)	
HER‐2 overexpression (%)^a^				
Positive	27 (20.8)	7 (24.1)	20 (19.8)	NS
Negative	77 (59.2)	21 (72.4)	56 (55.4)	
Unknown	26 (20.0)	1 (3.4)	25 (24.8)	

a DCIS cases were excluded during the analysis.

b Invasive ductal carcinoma.

c Ductal carcinoma in situ.

d Invasive lobular carcinoma.

e Lobular carcinoma in situ.

### Correlation between 3*β*‐HSD type 1 expression and clinical outcome in 130 ER‐positive breast cancers

**Figure 4:**
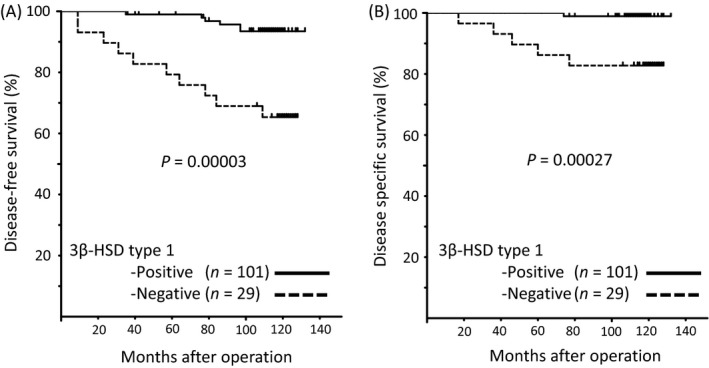
Disease‐free and disease‐specific survival curves of 130 ER‐positive breast cancers. Disease‐free (A) and disease‐specific (B) survival curves of 130 ER‐positive breast cancers according to 3*β*‐HSD type 1 expression were generated using the Kaplan–Meier method. The statistical significance of differences in the survival analyses were calculated using the log‐rank test. Significance was set as *P *<* *0.05.

Disease‐free survival curves of 130 ER‐positive breast cancers are illustrated in Figure 4A. 3*β*‐HSD type 1 expression was associated with a significant decreased risk of recurrence (log‐rank test; *P *=* *0.00003). After univariate analysis by Cox (Table 3), lymph node metastasis (*P *=* *0.006), histological grade 3 (*P *=* *0.003) and 3*β*‐HSD type 1‐negative (*P *=* *0.0003) were demonstrated to correlate significantly with worse prognosis for disease‐free survival in 130 ER‐positive breast cancer patients. Multivariate analysis (Table 3) revealed that histological grade 3 (*P *=* *0.020) and negativity of 3*β*‐HSD type 1‐negative (*P *=* *0.027) were independent prognostic factors with relative risks of 4.23 and 3.36, respectively, whereas the other factors described above were not significant.

**Table 3:** Univariate and multivariate analyses of disease‐free survival in 130 ER‐positive breast cancers

	Univariate*P*‐value	Multivariate	
		*P*‐value	Relative risk (95% CI)
Age	0.700		
Post‐menopausal state	0.475		
Histological type	0.525		
Invasive tumor size >20 mm	0.097		
Lymphatic involvement	0.108		
Lymph node metastasis	0.006^a^	0.080	3.23 (0.868–12.053)
Histological grade 3	0.003^a^	0.020	4.23 (1.252–14.266)
PgR LI ≥10%	0.320		
HER‐2 overexpression	0.799		
3*β*‐HSD type 1‐negative	0.0003^a^	0.027	3.36 (1.148–9.821)

a Data were considered significant by univariate analyses and examined by multivariate analyses.

Disease‐specific survival curves of 130 ER‐positive breast cancers are shown in Figure 4B. 3*β*‐HSD type 1‐positive cases showed significantly better prognosis compared with 3*β*‐HSD type 1‐negative cases (log‐rank test; *P *=* *0.00027). Using a univariate analysis (Table 4), histological grade 3 (*P *=* *0.038) and 3*β*‐HSD type 1‐negative (*P *=* *0.009) were identified as prognostic factors for disease‐specific survival. Multivariate analysis (Table 4) revealed that only 3*β*‐HSD type 1‐negative (*P *=* *0.023) was an independent prognostic factor, with relative risk of 12.23.

**Table 4:** Univariate and multivariate analyses of disease‐specific survival in 130 ER‐positive breast cancers

	Univariate*P*‐value	Multivariate	
		*P*‐value	Relative risk (95% CI)
Age	0.598		
Post‐menopausal state	0.523		
Histological type	0.482		
Invasive tumor size >20 mm	0.671		
Lymphatic involvement	0.209		
Lymph node metastasis	0.552		
Histological grade 3	0.038^a^	0.076	4.70 (0.852–25.912)
PgR LI ≥ 10%	NA^b^		
HER‐2 overexpression	0.656		
3*β*‐HSD type 1‐negative	0.009^a^	0.023	12.23 (1.418–105.369)

a Data were considered significant by univariate analyses and examined by multivariate analyses.

b Analysis was impossible because of multicollinearity.

## Discussion

In this study, expression of 3*β*‐HSD type 1 was inversely correlated with invasive tumor size, presence of invasive region, and lymphatic involvement. Moreover, our findings indicated that 3*β*‐HSD type 1 is a potent prognostic factor for better outcome in hormone‐dependent breast cancer.

In humans, there are two 3*β*‐HSD isoenzymes, which are designated as type 1 and type 2 and encoded by *HSD3B1* and *HSD3B2* genes, respectively 15. Type 1 isozyme is predominant in the placenta and peripheral tissues, such as the skin (principally in sebaceous glands), mammary gland, and prostate 29, 30, 31, 32. In comparison, the type 2 isozyme, which shares 93.5% identity with type 1, is almost exclusively expressed in the adrenal glands, ovary, and testis 29, 33, 34. In normal adrenal cortex, it is reported that 3*β*‐HSD type 1 immunoreactivity was essentially confined to the zona glomerulosa. In contrast, 3*β*‐HSD type 2 was not confined to the zona glomerulosa, but was found across the zona fasciculata 24. Based on these data, we used tissue sections of human placenta and human adrenal gland as positive controls for 3*β*‐HSD type 1 in this study, and our findings were consistent with previous studies. Furthermore, consistent with our expectations, immunoreactivity of 3*β*‐HSD type 1 was stronger in E10‐HSD3B1 cells compared with E10‐control cells. From these data, the antibody used in this study was considered to have sufficient specificity for 3*β*‐HSD type 1. Enzymatic activity of 3*β*‐HSD‐expressing tissue has been reported in human breast cancer tissues 35, and 3*β*‐HSD protein was observed in 36% of breast cancer samples 36. In mammary gland, sections immunolabeled for 3*β*‐HSD localization, labeling was observed in the cytoplasm of epithelial cells in both the acini and terminal ducts. Immunolabeling was also found in endothelial cells as well as in fibroblasts in the stroma and blood vessels 37. Our results do not necessarily coincide with previous reports in terms of the positive rate of 3*β*‐HSD in breast cancer tissues because of the different sample number and antibody used for 3*β*‐HSD detection. However, localization of 3*β*‐HSD type 1 in present study is in good agreement with previous studies.

In this study, multivariate analyses revealed that 3*β*‐HSD type 1‐negative is an independent prognostic factor, and that relative risks for disease‐free survival and disease‐specific survival were 3.36 and 12.23, respectively. These data suggest that the effectiveness of 3*β*‐HSD type 1 as a prognostic marker for is at least equal or higher than other prognostic markers previously reported, which include invasive tumor size, lymph node status, histological grade, PgR status, and HER‐2 status 38, 39. However, prospective studies are needed to clarify whether 3*β*‐HSD type 1 can be used as a new prognostic marker of breast cancers in routine practice. The current view is that inhibition of 3*β*‐HSD1 would decrease conversion of DHEA to estrogen precursors or DHT to 3*β*‐diol, to slow ER‐positive tumor growth 40. In our previous report, we suggest that increased expression of HSD3B1 might reduce sensitivity to aromatase inhibitors (AIs) in human breast cancer cell lines, as demonstrated by enhanced 3*β*‐diol‐induced ER activation and growth mechanisms 25. Another study suggested that the steroid‐metabolizing pathway activated by 3*β*‐HSD type 1 might function as an alternative estrogenic steroid‐producing aromatase‐independent pathway in human breast cancers 41. Therefore, we initially focused on the steroid‐metabolizing pathway of 3*β*‐HSD type 1 as a tumor progression factor, or one candidate of the AI‐resistance mechanism. However, in this study, expression of 3*β*‐HSD type 1 was inversely correlated with invasive tumor size, presence of invasive region, and lymphatic involvement. Moreover, it was associated with a decreased risk of recurrence in cases that were treated with AI as an adjuvant therapy (*n* = 44; data not shown), and this result was inconsistent with our previous report. In this study, we found a significant positive correlation between ER positivity and 3*β*‐HSD type 1 expression. We already know that ER+ breast cancers have better outcomes. Therefore, it is suggested that expression of 3*β*‐HSD might just indirectly indicate the hormone‐responsiveness of individual breast cancer cases. Further analyses including validation using other breast cancer databases or in vitro experiments are required to resolve this paradox and to understand the function of 3*β*‐HSD type 1 in breast cancer.

**Figure 5:**
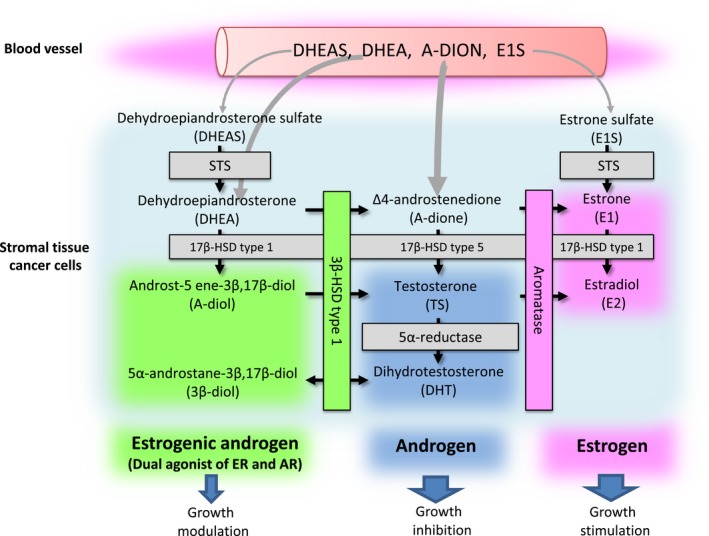
Steroid metabolism in breast cancer tissues. Bioactive sex steroids are locally produced from circulating inactive steroids including dehydroepiandrosterone sulfate (DHEAS), dehydroepiandrosterone sulfate (DHEA) and androstenedione (A‐dione) by the action of the various steroid‐metabolizing enzymes. Aromatase catalyzes the final and rate‐limiting step in the biosynthesis of estrogen from adrenal androgens (testosterone [TS] and A‐dione). Steroid sulfatase (STS) metabolizes estrone sulfate (E1S) and DHEAS to E1 and DHEA, respectively, which are further metabolized to E2 or androst‐5‐ene‐3*β*,17*β*‐diol (A‐diol), respectively, by 17*β*‐hydroxysteroid dehydrogenase type 1 (17*β*‐HSD type 1). dihydrotestosterone (DHT) is synthesized from TS in an irreversible reaction catalyzed by the 5*α*‐reductase family, and is a highly potent androgen. Human 3*β*‐HSD type 1 is a critical enzyme in the conversion of DHEA and A‐diol to estrogen precursors A‐dione and TS. This enzyme is also involved in conversion of the potent androgen DHT to its inactive form 5*α*‐androstane‐3*β*,17*β*‐diol (3*β*‐diol), which has substantial estrogenic activity.

As mentioned above, bioactive sex steroids are locally produced from circulating inactive steroids by the comprehensive action of the various steroid‐metabolizing enzymes (Fig. 5). AIs are established as a standard treatment option for hormone‐dependent breast cancer. It is suggested that STS‐17*β*‐HSD type 1 pathway could function as another estrogenic steroid‐producing pathway 41. Indeed, the clinical and prognostic significance of STS in human breast cancer has been reported 28. Furthermore, the STS pathway has been noted as a therapeutic target, and its clinical application is already underway 12, 42. Expression of the 5*α*‐reductase family in the breast has been reported to be associated with better prognosis in breast cancer patients 43, 44. Thus, many suggestions for therapeutic targets or biological markers of hormone‐dependent breast cancer have been generated through understanding the intracrine systems of breast cancer. Further understanding of 3*β*‐HSD type 1 may provide insights for the development of novel therapeutic strategies in breast cancer treatment.

In this analysis, we have not studied 3*β*‐HSD type 1 with respect to AR expression. However, it is necessary to extend the analysis to its association with AR expression in the future because this enzyme is an androgen‐metabolizing enzyme. The role of 3*β*‐HSD type 1 in ER‐negative breast cancer was not sufficiently examined because of the small number of cases. Of 31 ER‐negative cases, the number of cases evaluated as 3*β*‐HSD type 1‐positive and ‐negative were 18 (58.1%) and 13 (41.9%), respectively. Although there was no statistical significance, 3*β*‐HSD type 1 expression tended to be inversely correlated with invasive tumor size, presence of invasive region, lymphatic involvement, and lymph node metastasis. Therefore, a possibility that the function of 3*β*‐HSD type 1 is independent of ER must also be considered. As mentioned above, prospective studies with a larger number of cases are needed in the future to promote our further understanding of 3*β*‐HSD type 1.

In summary, we have investigated the clinical and prognostic significance of 3*β*‐HSD type 1, which may regulate in situ production or inactivation of active sex steroids in hormone‐dependent breast cancers using IHC. This is the first study to indicate that 3*β*‐HSD type 1 is a potent prognostic factor in hormone‐dependent breast cancer.

## Conflict of Interest

The authors declare that they have no conflict of interest.
